# Identification of diagnostic signature, molecular subtypes, and potential drugs in allergic rhinitis based on an inflammatory response gene set

**DOI:** 10.3389/fimmu.2024.1348391

**Published:** 2024-02-26

**Authors:** Jun Dai, Keyu Xia, De Huai, Shuo Li, Lili Zhou, Shoufeng Wang, Li Chen

**Affiliations:** ^1^Department of Otorhinolaryngology, The Affiliated Huai’an Hospital of Xuzhou Medical University and The Second People’s Hospital of Huai’an, Huai’an, Jingsu, China; ^2^Department of Otorhinolaryngology, The Fifth People’s Hospital of Huai’an, Huai’an, Jingsu, China; ^3^Department of Otorhinolaryngology, People’s Hospital of Hongze District, Huai’an, Jingsu, China

**Keywords:** allergic rhinitis, diagnostic biomarkers, subtypes, inflammatory response, molecular docking

## Abstract

**Background:**

Rhinitis is a complex condition characterized by various subtypes, including allergic rhinitis (AR), which involves inflammatory reactions. The objective of this research was to identify crucial genes associated with inflammatory response that are relevant for the treatment and diagnosis of AR.

**Methods:**

We acquired the AR-related expression datasets (GSE75011 and GSE50223) from the Gene Expression Omnibus (GEO) database. In GSE75011, we compared the gene expression profiles between the HC and AR groups and identified differentially expressed genes (DEGs). By intersecting these DEGs with inflammatory response-related genes (IRGGs), resulting in the identification of differentially expressed inflammatory response-related genes (DIRRGs). Afterwards, we utilized the protein–protein interaction (PPI) network, machine learning algorithms, namely least absolute shrinkage and selection operator (LASSO) regression and random forest, to identify the signature markers. We employed a nomogram to evaluate the diagnostic effectiveness of the method, which has been confirmed through validation using GSE50223. qRT-PCR was used to confirm the expression of diagnostic genes in clinical samples. In addition, a consensus clustering method was employed to categorize patients with AR. Subsequently, extensive investigation was conducted to explore the discrepancies in gene expression, enriched functions and pathways, as well as potential therapeutic drugs among these distinct subtypes.

**Results:**

A total of 22 DIRRGs were acquired, which participated in pathways including chemokine and TNF signaling pathway. Additionally, machine learning algorithms identified NFKBIA, HIF1A, MYC, and CCRL2 as signature genes associated with AR’s inflammatory response, indicating their potential as AR biomarkers. The nomogram based on feature genes could offer clinical benefits to AR patients. We discovered two molecular subtypes, C1 and C2, and observed that the C2 subtype exhibited activation of immune- and inflammation-related pathways.

**Conclusions:**

NFKBIA, HIF1A, MYC, and CCRL2 are the key genes involved in the inflammatory response and have the strongest association with the advancement of disease in AR. The proposed molecular subgroups could provide fresh insights for personalized treatment of AR.

## Introduction

Rhinitis, affecting over 400 million individuals globally, is a prevalent condition caused by allergens that triggers an inflammatory response. It is characterized by symptoms such as the presence of postnasal drip, frequent sneezing, nasal congestion, and excessive nasal discharge (1, 2). There are three main types of rhinitis: infectious rhinitis, allergic rhinitis (AR), and nonallergic rhinitis (NAR), which can be further categorized into distinct phenotypes (3). AR, which includes perennial allergic rhinitis and seasonal allergic rhinitis, is the most common form of rhinitis (4). AR is a type of atopic disease that is mediated by immunoglobulin E (IgE) (5). It is characterized by symptoms such as congestion, sneezing, and itching in the nose, all of which have a negative impact on the individual’s quality of life (6). Studies have previously demonstrated that the occurrence of AR in adults in China has risen significantly, with the prevalence increasing from 11.1% to 17.6% (7). AR is often accompanied by other health conditions like asthma and allergic conjunctivitis (8, 9). Indeed, research has indicated that clinical asthma is present in 20% to 50% of individuals with AR, while over 80% of patients diagnosed with allergic asthma also experience symptoms of rhinitis simultaneously (10). In addition, individuals suffering from AR frequently experience reduced productivity in learning and work, disrupted sleep patterns, diminished quality of life, and in some cases, psychological conditions like depression. As a result, this places a significant financial strain on society (11, 12). Despite significant progress in comprehending the pathophysiology of AR, its early detection, treatment intervention, and underlying causes continue to pose challenges. Consequently, there is an immediate need to further explore the pathogenesis of AR in order to identify effective targets for therapeutic interventions.

Rhinitis is a diverse condition that has been linked to inflammatory reactions, as seen in AR, but can also manifest without inflammation, as observed in idiopathic rhinitis (13). The development of AR involves a complex and diverse set of causes and factors. AR is characterized by the involvement of immune inflammation and IgE, which are crucial in driving the allergic inflammatory process (14). The inflammatory response in AR is distinguished by the entrance of inflammatory cells, such as eosinophils, basophils, mast cells, and T cells, into the nasal mucosa (15, 16). Overexpression of IL-36γ amplifies eosinophilic inflammation in AR by enhancing the adhesion, survival, and activation of eosinophils (17). Moreover, targeting the NLRP3 inflammasome-induced pyroptosis pathway holds great potential as a viable therapeutic approach to alleviate inflammatory reactions in individuals with AR (18). Earlier research has demonstrated that a correlation exists between inflammatory response-related genes (IRRGs) and various illnesses, such as diabetic kidney disease and sepsis (19, 20). As a result, the identification of diagnostic and therapeutic targets linked to inflammatory response is anticipated to impede the progression of the AR. This study aims to identify inflammation-related diagnostic biomarkers for patients with AR, making it a valuable contribution to the field.

## Methods

### Acquisition and pre-processing of raw data

The Gene Expression Omnibus (GEO) database is an extensive public resource for gene expression data across various species (21). We obtained the transcriptome data from the GEO database, which can be accessed at https://www.ncbi.nlm.nih.gov/geo/. For our study, the datasets must satisfy the following criteria: 1) The AR group should comprise at least 20 participants; 2) The research must focus on Homo sapiens; 3) The raw or processed data must be publicly available; 4) The research must involve blood samples from both individuals with AR and healthy individuals. The datasets used in this study consisted of the following: GSE75011, which included 15 healthy control (HC) samples and 25 samples from individuals with AR, and GSE50223, which included 21 HC samples and 21 AR samples. GSE75011 was chosen as the test set, while GSE50223 was selected as the validation set. The data obtained from these GEO datasets underwent preprocessing and normalization using the “affy” R package.

### Identifying differentially expressed genes in patients with AR

Using the limma package, we performed a differential expression analysis on the GSE75011 dataset, comparing the HC and AR groups. The DEGs were determined based on the criteria of a p-value < 0.05 and a |log-fold change (FC)| > 0.5.

### Identifying differentially expressed inflammatory response-related genes and performing functional enrichment analysis on them

To determine the most significant pathways between the HC and AR groups, we utilized the ClusterProfiler package to perform Gene Set Enrichment Analysis (GSEA). We obtained the “h.all.v7.4.symbols.gmt” subset from the Molecular Signatures Database to assess pertinent pathways. Statistical significance was defined as a p value below 0.05. Subsequently, the gene set variation (GSVA) method was employed to assess the inflammatory score. To accomplish this, we employed the GSVA package to calculate the inflammatory response score for each sample within the inflammatory response gene set. The resulting inflammatory response score was then visually represented using a boxplot. We obtained a total of 200 inflammatory response-related genes (IRGGs) from the MSigDB (inflammatory response gene set). To obtain the gene expression profile of DIRGGs, we intersected these IRGGs with the DEGs identified from the GSE75011 dataset. By utilizing the “pheatmap” R package, we generated a heat map that illustrates the expression patterns of DIRGGs. We used the ClusterProfiler package to perform functional enrichment analyses on DIRRGs. Our analysis involved the use of the Kyoto Encyclopedia of Genes and Genomes (KEGG) and Gene Ontology (GO). GO function annotation includes three categories: biological process (BP), cell component (CC), and molecular function (MF). The statistical significance was determined by enrichment, with a significance level set at p < 0.05. Ultimately, the results were visually represented using the ggplot2 package.

### Construction of protein-protein interaction network

The STRING database (https://string-db.org/) is a valuable resource that allows users to investigate protein interactions (22). We rely on the STRING database to construct PPI networks, with a confidence score of 0.4 serving as the threshold for determining the reliability of these interactions. CytoHubba offers 11 different topological analysis techniques, encompassing Maximal Clique Centrality Degree (MCC), Density of Maximum Neighborhood Component, Maximum Neighborhood Component, Edge Percolated Component, and six centralities (Stress, Betweenness, Radiality, Closeness, EcCentricity, and Bottleneck). Out of these eleven methods, the recently introduced MCC method demonstrates superior precision in predicting essential proteins from the PPI network, setting it apart from the rest (23). We utilized the cytoHubba plugin within the Cytoscape software to identify hub genes using the MCC method. The top 10 genes with the highest MCC scores were chosen as hub genes, and these selected genes were further investigated in subsequent analyses (24).

### Random forest and LASSO algorithms are utilized to screen signature genes

To identify potential candidate genes for diagnosing AR, two machine learning algorithms were used. The randomForest package in R was used to construct a model and calculate the average error rate for all DIRRGs. Another random forest (RF) model was created to determine importance values by reducing accuracy. Genes with an importance value > 1 were identified as hub genes for future model development (25). The top 8 genes were chosen as promising candidates. The glmnet package was used for LASSO analysis, with penalty parameters for 10-fold cross-validation. This approach surpasses traditional regression analysis for high-dimensional data assessment (26). Subsequently, we obtained diagnostic genes by identifying the genes based on their scores in MCC, RF, and LASSO, and then intersecting the resulting gene lists.

### Construction and evaluation of nomogram model

The construction of a nomogram is extremely advantageous for the diagnosis of clinical AR. To create the nomogram, we employed the rms R package and considered the candidate genes (27). Each candidate gene was given a score known as “points”, while the “Total Points” represents the total score of all the genes. The diagnostic efficacy of both the candidate genes in AR diagnosis was evaluated by establishing a ROC curve. Additionally, the model’s accuracy was assessed using decision curve analysis (DCA) and calibration curves.

### Single gene GSEA

To conduct single gene GSEA analysis, we utilized the GSEA software to classify the samples into high and low expression groups, using the median values of diagnostic gene expression levels as the criteria. In order to explore the underlying molecular mechanisms associated with gene phenotypes, we obtained the “c2.cp.kegg.v7.4.symbols.gmt” subset from the Molecular Signatures Database. The minimum gene set was set to 5, while the maximum gene set was set to 5000. Additionally, one thousand resamplings were performed. A p value lower than 0.05 was considered to be statistically significant.

### Analysis of the immune microenvironment

The ssGSEA algorithm utilized immune gene sets that incorporated genes associated with various immune cell checkpoints, pathways, functions, and types. To comprehensively evaluate the immunological attributes of each sample, we applied the ssGSEA algorithm using R packages such as limma, GSEABase and GSVA (28). Furthermore, the ggplot2 package was utilized to investigate the association between the expression of diagnostic genes and the infiltration of immune cells. The findings were visually presented in a lollipop chart.

### Identification of different AR subtypes using unsupervised clustering

The ConsensusClusterPlu package was employed in the study to conduct unsupervised cluster analysis using the expression profile of 22 DIRGGs. This facilitated the categorization of AR patients into distinct subgroups and the identification of the most suitable number of clusters.

### Identification of small-molecule drugs

The Connectivity Map (CMap) is a database of expression profiles that harnesses cellular responses to disturbances in order to uncover potential functional connections between treatments, genes, and diseases (29). In this study, we performed CMap analysis to anticipate small-molecule compounds that target subtypes associated with inflammatory response. We extracted drug signatures from the Connectivity Map database (CMap, https://clue.io/). Our input data consisted of the top 100 up-regulated and 100 down-regulated genes in the two subtypes. The final analysis result is assigned a score ranging from -100 to 100. In the case of small-molecule drugs, a negative score suggests the ability to reverse gene expression, which signifies their potential therapeutic significance. We chose the top five small-molecule compounds based on their lowest CMap score.

### Molecular docking analysis

The binding affinity between signature gene proteins and small-molecule drugs was investigated by molecular docking technique (30). The protein structure of signature genes was obtained from the PDB database, while the molecular structures of small-molecule drugs were acquired from PubChem. Ligands and receptors were then processed using AutoDockTools, primarily through the addition of hydrogen atoms and identification of active pockets. The binding modes between the candidate protein and small-molecule drugs were subsequently analyzed using AutoDock Vina software.

### Quantitative real-time PCR analysis

A total of 16 individuals participated in the study, with an equal distribution of 8 HC and 8 patients diagnosed with AR. The blood samples were collected from the Affiliated Huai’an Hospital of Xuzhou Medical University. Prior to the collection, all participants provided written informed consent, and the study protocol received approval from the Ethics Committee of the Affiliated Huai’an Hospital of Xuzhou Medical University (ethical approval number: 2022029). We collected peripheral blood in tubes containing citrate, which were then stored at 4°C until ready for use.

RNA was extracted from blood samples using the TRIzol reagent (Invitrogen, CA, USA) following the manufacturer’s instructions. Then, the RNA underwent spectrophotometric quantification at a wavelength of 260 nm, and its purity was assessed by determining the absorbance ratio at 260/280 nm. The absorbance ratios (260/280 nm) of the RNA samples are fall within the range of 1.8 to 2.1. 2 μg of RNA was then subjected to reverse transcription using the First Strand cDNA Synthesis Kit (Invitrogen, CA, USA). qRT-PCR analysis was performed using the SYBR qPCR Master Mix (Sigma, MO, USA). The parameter settings during amplification are as follows: initial denaturation at 95°C for 10 minutes, followed by 40 cycles consisting of denaturation at 95°C for 10 seconds, annealing at 60°C for 30 seconds, extension at 72°C for 1 second, and final cooling at 40°C for 30 seconds. The expression levels of the diagnostic signature genes were measured using the Roche LC480 Real-Time PCR System (Roche). The internal control for mRNA was β-actin. The primers were presented in **Supplementary Table S1**.

## Results

### Expression profile of DIRRGs in AR

**Figure 1:**
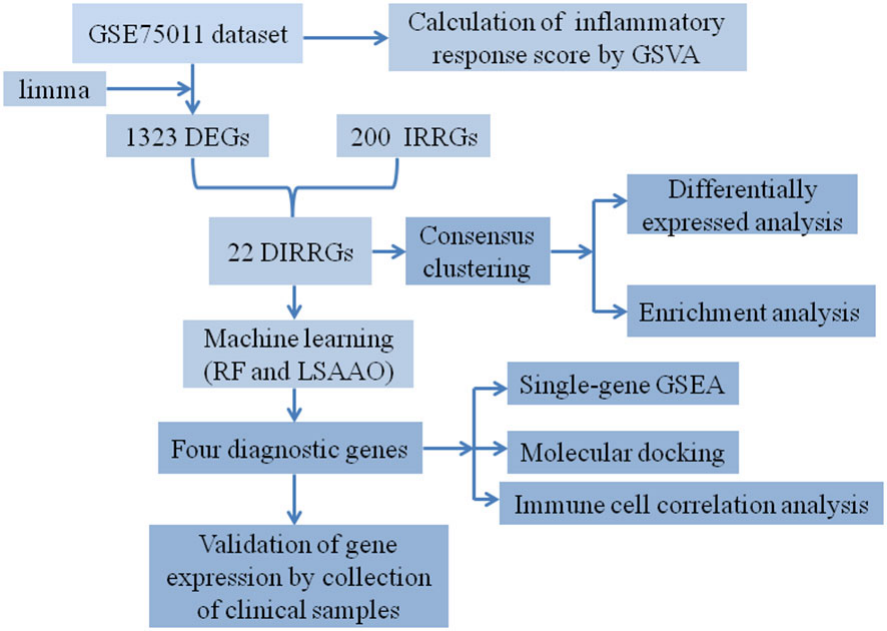
The workflow diagram of our study.

**Figure 2:**
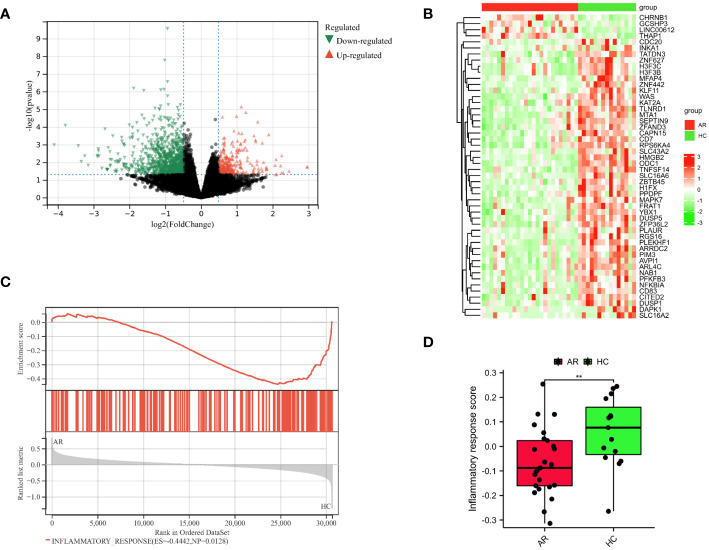
Identification of DEGs in AR. **(A)** Diagrams of the DEGs between the HC and AR groups are represented by volcano plots. **(B)** Heatmap plots are used to visualize the top 50 DEGs between the HC and AR groups. **(C)** GSEA showed inflammatory response plays a vital role in the development of AR. **(D)** Boxplots demonstrated variations in the inflammatory response score between the AR and HC groups. **p < 0.01. These results showed that the development of AR is closely linked to the inflammatory response.

The flowchart illustrating the analysis process for this study is presented in **Figure 1**. A comparative analysis was conducted between samples from HC and AR, resulting in the identification of 1323 DEGs. Among these DEGs, 307 were upregulated, whereas 1016 were found to be downregulated (**Figure 2A**). The heat map displayed the level of DEGs in the top 50 between the two groups. The majority of these genes exhibit a downward trend (**Figure 2B**). The GSEA analysis revealed a significant enrichment of the inflammatory response gene set in the HC group (**Figure 2C**). Moreover, the GSVA algorithm was utilized to calculate the inflammatory response score, highlighting a significantly lower score in the AR group compared to the HC group (**Figure 2D**). Based on our findings, it becomes evident that the development of AR is closely associated with the inflammatory response. Therefore, our study aims to delve deeper into the role of IRGGs in AR, shedding light on their significance in this process.

### Functional enrichment analysis

**Figure 3:**
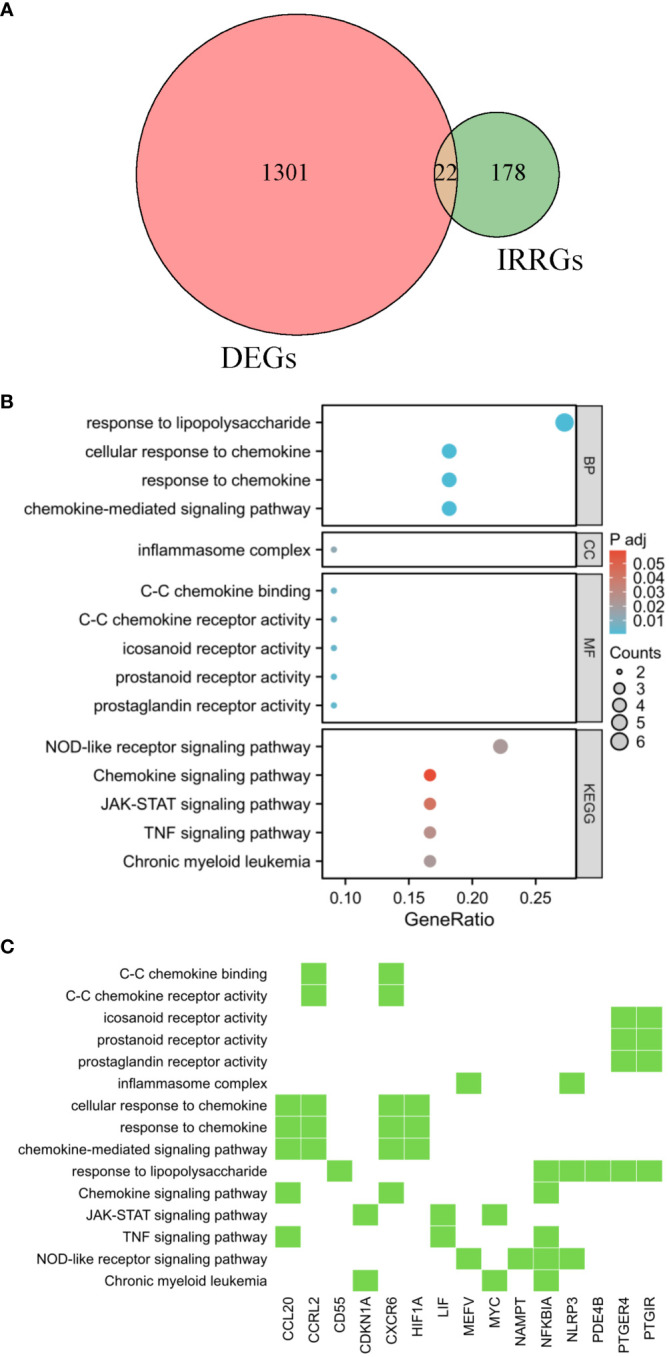
Functional enrichment analysis. **(A)** The shared genes between the DEGs and IRRGs were represented in the Venn diagram. GO and KEGG results are presented as bubble plots **(B)** and heat maps **(C)**. The Venn diagram, bubble chart, and heatmap provide a comprehensive view of the potential biological functions and pathways associated with the genes at the intersection of DEGs and IRRGs, offering insights into their roles in AR pathogenesis.

Enrichment analysis was performed on the overlapping genes between DEGs and IRRGs. In this study, we identified a total of 22 DIRRGs, as shown in **Figure 3A**. Our findings demonstrated that the majority of these DIRRGs, such as PLAUR, NFKBIA, RGS16, GPR132, PTGIR, HIF1A, CXCR6, CDKN1A, LIF, CD55, PDE4B, GNA15, PTGER4, NAMPT, NLRP3, CCRL2, ICOSLG, and CCL20, exhibited a significant decrease in samples from AR patients compared to those from HC, as illustrated in **Supplementary Figure S1**. According to the data presented in **Figures 3B, C**, the results of the GO analysis revealed that these genes were significantly associated with BP, including response to lipopolysaccharide, cellular response to chemokine, and chemokine-mediated signaling pathway. Additionally, in terms of CC ontology, these genes were found to be involved in the inflammasome complex. Moreover, the MF analysis identified C-C chemokine binding, C-C chemokine receptor activity, and icosanoid receptor activity as significant terms for these genes. Furthermore, the KEGG analysis demonstrated that these genes were associated with pathways such as NOD-like receptor signaling pathway, chemokine signaling pathway, TNF signaling pathway, etc. We have also identified key players involved in the development of chronic myeloid leukaemia - CDKN1A, MYC and NFKBIA. These genes also intersect with key signaling pathways that control inflammatory responses and cell communication. In particular, the genes MEFV, NAMPT, NFKBIA and NLRP3 were involved in the NOD-like receptor signaling pathway, which orchestrates the body’s innate immunity. Meanwhile, CCL20, LIF and NFKBIA emerged as components of the TNF signalling pathway, a key mediator of inflammation and apoptosis. Similarly, the JAK-STAT pathway, a conduit for a variety of cytokines and growth factors, was influenced by the activities of CDKN1A, LIF and MYC. Finally, the chemokine signaling pathway, a key player in directing immune cell traffic, involves CCL20, CXCR6 and NFKBIA. Each of these pathways, shown in **Figure 3C**, reveals a complex tapestry of genetic interactions that are critical to our understanding of the mechanisms of AR.

### Employing machine learning algorithms to detect the potential biomarkers

**Figure 4:**
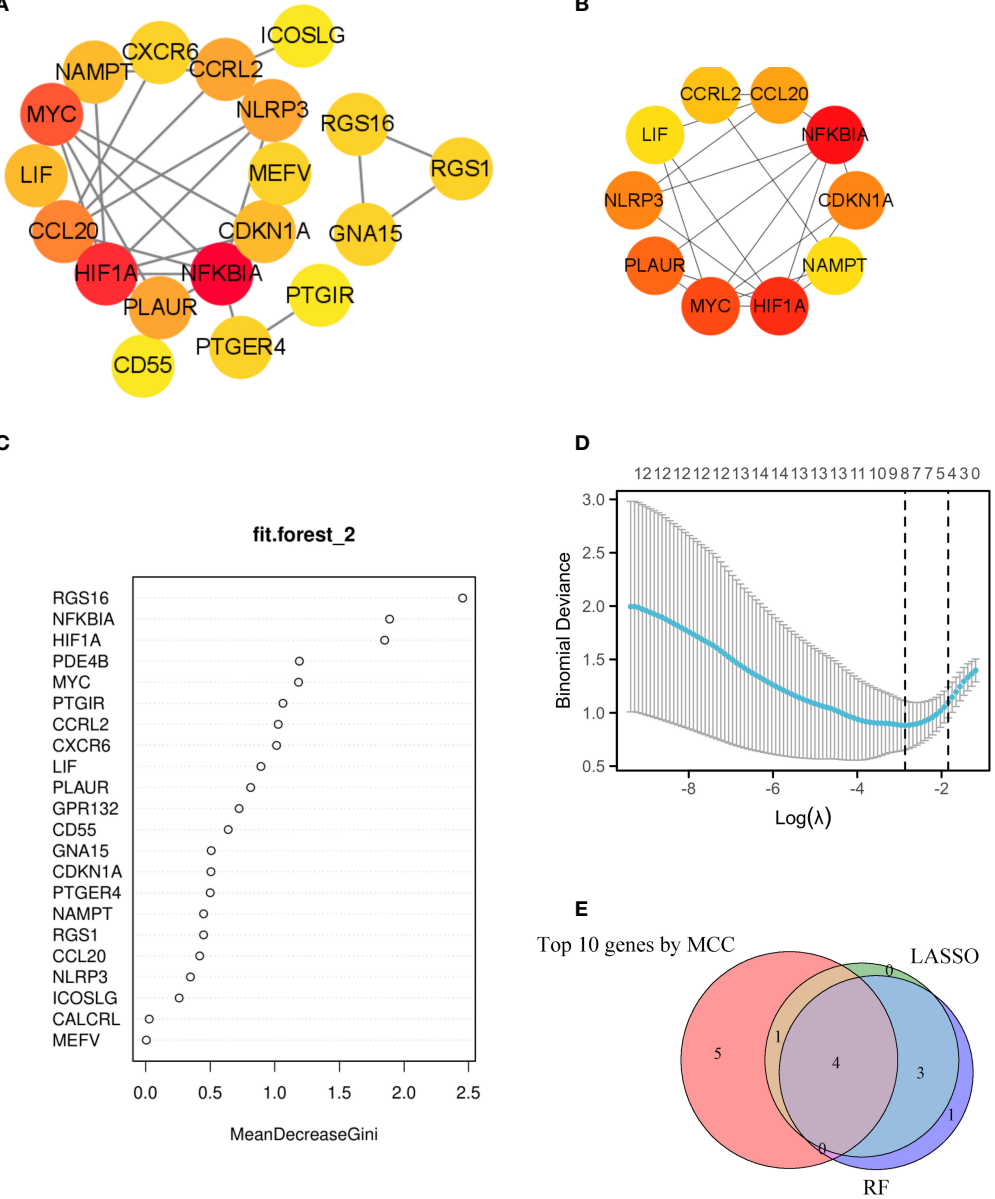
Utilizing machine learning algorithms for the identification of markers. **(A)** The interaction of 19 genes can be observed in the PPI network. **(B)** The top 10 genes with highest MCC scores were selected as hub genes for further investigation. **(C)** In the random forest model, genes exhibit a level of importance that exceeds zero. **(D)** The LASSO model has successfully identified key genes, with a total of 8 genes that are highly suitable for diagnostic purposes. **(E)** The Venn diagram showed 4 diagnostic genes identified using the mentioned machine learning techniques and MCC. The machine learning algorithms could pinpoint crucial genes implicated in inflammatory response, laying the groundwork for potential biomarker identification and targeted therapeutic strategies.

A PPI network was established, illustrating the interaction between 19 different genes (**Figure 4A**). The ranking of these genes based on their MCC score can be observed in **Figure 4B**. To identify signature genes in patients with AR, two machine algorithms were employed. The random forest analysis successfully identified 9 signature genes with a relative importance exceeding 1, as demonstrated in **Figure 4C**. Additionally, the LASSO analysis specifically selected 8 signature genes, as illustrated in **Figure 4D**. **Figure 4E** displayed a Venn diagram depicting the overlap between the top 10 genes identified using MCC, the 8 significant genes identified using RF, and the 8 potential candidate genes identified using LASSO. For analysis and validation purposes, four signature genes (NFKBIA, HIF1A, MYC, CCRL2) were identified from this intersection.

### Development of the nomogram and evaluation of its diagnostic value

**Figure 5:**
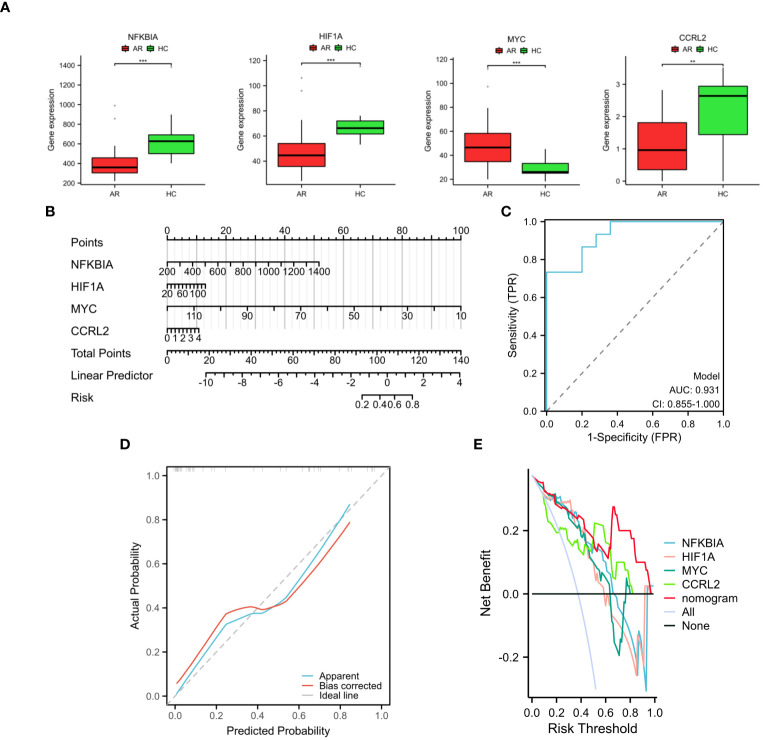
Development and validation of the nomogram. **(A)** The expression of four diagnostic genes in the GSE75011 dataset. **(B)** Nomogram used to assess AR development potential. **(C)** Nomogram’s ROC curve for diagnosing AR. **(D)** Nomogram’s calibration curve. **(E)** Decision curve analysis with nomogram model. These findings showed the potential utility of the selected genes as biomarkers for AR and underscore the efficacy of the proposed predictive model in clinical diagnosis. **p < 0.01, ***p < 0.001.

In the AR group, the levels of NFKBIA, HIF1A, and CCRL2 expression were lower compared to the HC group. Conversely, the mRNA expression of MYC was higher in the AR group than in the HC group (**Figure 5A**). In order to improve the accuracy of predicting the development of AR patients, a nomogram has been created which incorporates the analysis of four specific genes (**Figure 5B**). By analyzing the receiver operating characteristic (ROC) curve, it was observed that the model performed exceptionally well, with a significant area under the curve (AUC) value of 0.931 (**Figure 5C**). The results from the calibration curve (**Figure 5D**) further validated the remarkable precision of the nomogram model in predicting outcomes for patients with AR. Moreover, the decision curve analysis demonstrated the potential advantages of employing the nomogram model in patients with AR, as shown in **Figure 5E**. Moreover, the model’s reliability was assessed by using an external validation dataset (GSE50223) (**Supplementary Figure S2**). The results demonstrated that the nomogram achieved a remarkable diagnostic accuracy for AR, highlighting its efficacy.

**Figure 6:**
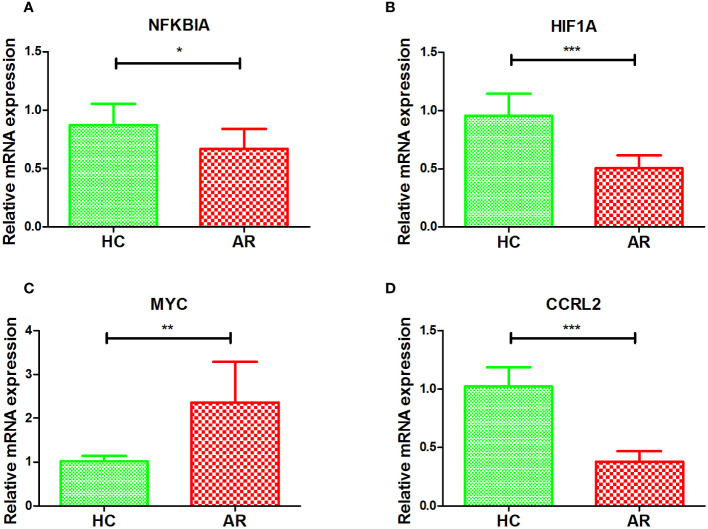
Validation of NFKBIA **(A)**, HIF1A **(B)**, MYC **(C)**, and CCRL2 **(D)** by collected clinical samples. *p < 0.05, **p < 0.01, ***p < 0.001.

To further validate the diagnostic value of the four markers in diagnosing AR, we gathered clinical blood samples. With these samples, we aimed to confirm the effectiveness of these markers in accurately identifying AR cases. In **Figures 6A–D**, the AR group had lower expression of NFKBIA, HIF1A, and CCRL2 compared to HC, while the AR group had higher expression of MYC (p < 0.05 or p < 0.01 or p < 0.001). Based on our research, we discovered that the above-mentioned genes associated with the inflammatory response exhibit a strong diagnostic capability, making them potential biomarkers for diagnosing AR.

### Exploring possible biological roles of diagnostic DIRRGs

**Figure 7:**
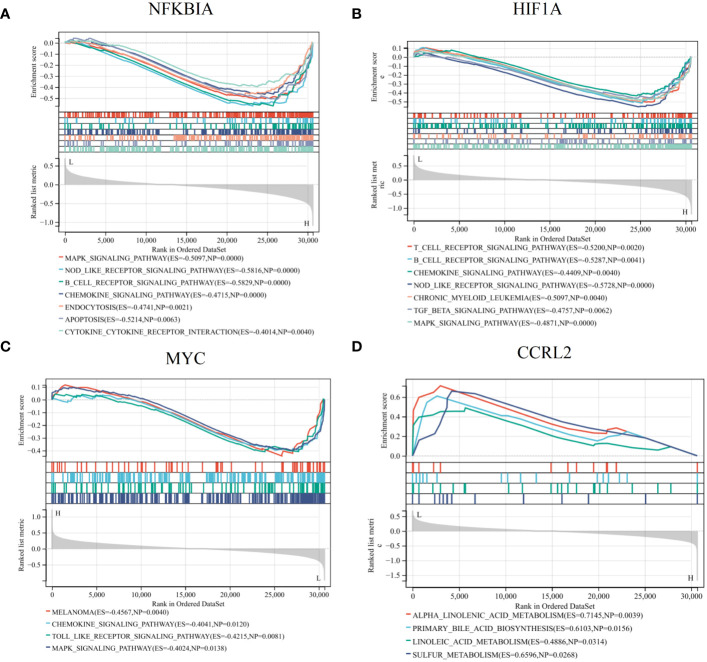
GSEA revealed the potential pathways linked to the diagnostic genes. Single gene GSEA of NFKBIA **(A)**, HIF1A **(B)**, MYC **(C)**, CCRL2 **(D)** in AR. These findings provided insights into the biological processes and pathways that may be modulated by the expression of these key genes, potentially unveiling their mechanistic roles in AR pathogenesis or progression.

We conducted a detailed examination of potential signaling pathways linked to signature genes using GSEA. The results obtained from single gene GSEA indicated that the NFKBIA high-expressed phenotype was enriched with MAPK signaling pathway, NOD like receptor signaling pathway, B cell receptor signaling pathway, chemokine signaling pathway, endocytosis, and apoptosis (**Figure 7A**); the HIF1A high-expressed phenotype was enriched with T cell receptor signaling pathway, B receptor signaling pathway, chemokine signaling pathway, chronic myeloid leukemia, TGF beta signaling pathway (**Figure 7B**); the MYC low-expressed phenotype was enriched with chemokine signaling pathway, toll like receptor signaling pathway, and MAPK signaling pathway (**Figure 7C**); the CCRL2 low-expressed phenotype was enriched with alpha linolenic acid metabolism, primary bile acid biosynthesis, linoleic acid metabolism, and sulfur metabolism (**Figure 7D**). These findings provided additional evidence that the NFKBIA, HIF1A, and MYC genes are all intricately connected to the immune system and the body’s response to inflammation.

### The outcomes of the infiltration of immune cells

**Figure 8:**
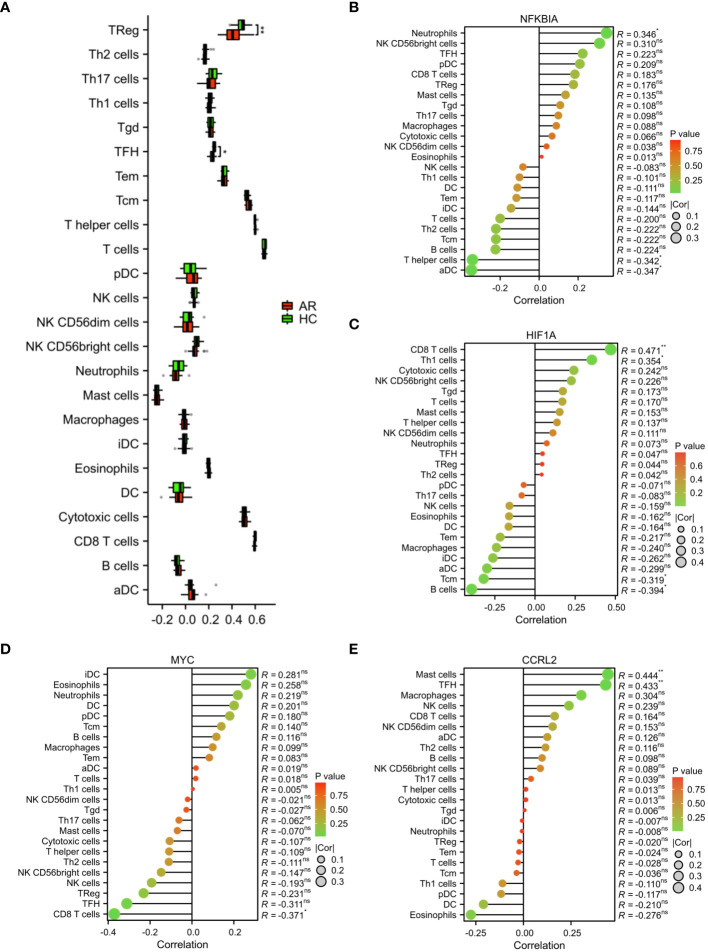
Analysis of immune cell infiltration. **(A)** Evaluation of the presence of immune cells in the AR and HC groups. **(B)** Assessment of the correlation between immune cells and the expression of signature genes. *p < 0.05, **p < 0.01. These correlation analyses provided insight into the potential regulatory roles of NFKBIA, HIF1A, MYC, and CCR2L in shaping the immune landscape in AR, offering opportunities for novel therapeutic targets and diagnostic markers. ns, no statistical difference.

In addition, we employed the ssGSEA algorithm to quantify the enrichment scores of immune cells in the blood of AR. The results showed a notable disparity in immune cell infiltration between the HC and AR groups, with the HC group exhibiting significantly higher levels of Treg and TFH cells (**Figure 8A**). By analyzing the correlation between immune cells, we have identified certain genes that may be involved in the progression of AR. These genes exert their influence by regulating immune cells like CD8 T cells, mast cells, neutrophils, B cells, and THF (**Figures 8B-E**). In conclusion, our study provided valuable insights into the underlying mechanisms of AR and the role of immune cell infiltration in its progression. The identification of signature genes associated with specific immune cells not only enhances our understanding of the AR but also opens up new avenues for targeted therapeutic interventions. Further research is needed to validate these findings and explore the potential clinical applications in the management of AR.

### Identifying subtypes of inflammation with different molecular mechanisms

**Figure 9:**
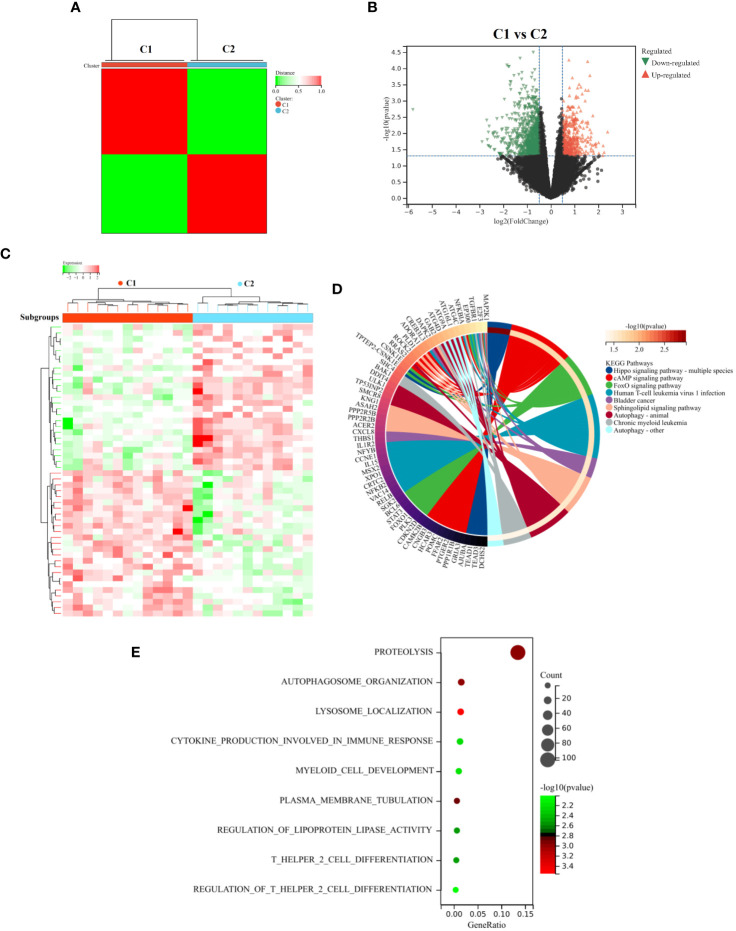
Identify and analyze subtypes of diseases related to inflammation. **(A)** Unsupervised cluster analysis. **(B)** The Volcano diagram and **(C)** Heatmap diagram represent the DEGs between the C1 and C2 subgroups. The results of KEGG **(D)** and GO **(E)** analysis between the two subtypes. These analyses enabled the visualization of distinct molecular characteristics and biological processes associated with the defined subgroups, providing insights into the mechanistic differences underlying these classifications.

We also classified AR patients into different inflammatory phenotypes using unsupervised cluster analysis. This allows us to better understand the variation and characteristics within the patient population. Based on the expression profiling of 22 DIRGGs, a consensus clustering approach was used to cluster AR patients. The results revealed that the optimal number of subtypes was determined to be 2 (**Figure 9A**). Notably, significant heterogeneity in gene expression was observed between these two subtypes. A comparative analysis of samples from subgroups C1 and C2 found a total of 1463 differentially expressed genes. Among these, 463 genes were observed to be upregulated, while 1000 genes were observed to be downregulated (**Figures 9B, C**). Furthermore, the results of KEGG and GO analysis indicated that these differentially expressed genes were predominantly enriched in pathways related to immune and inflammatory processes. These pathways encompassed autophagy, infection by human T cell leukemia virus 1, chronic myeloid leukemia, proteolysis, cytokine production involved in immune response, myeloid cell development, T helper 2 cell differentiation, and other related pathways (**Figures 9D, E**).

**Figure 10:**
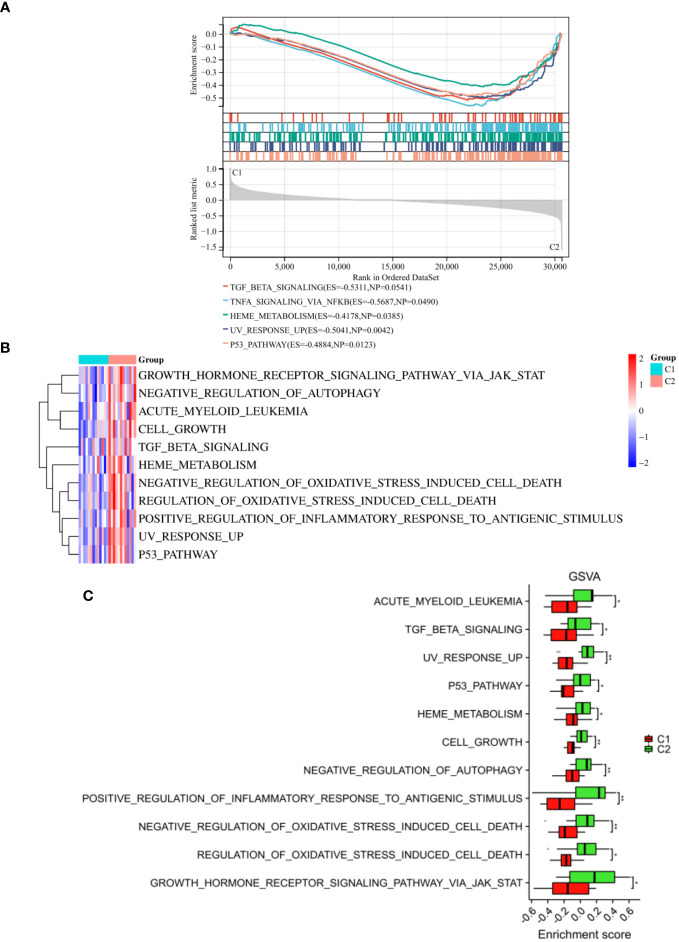
Enrichment analysis between the two subtypes. **(A)** The outcomes of GSEA were depicted. The results of GSVA were visualized through a heatmap **(B)** and a histogram **(C)**. *p < 0.05, **p < 0.01. These findings facilitated the understanding of the differential pathway between molecular subgroups C1 and C2, contributing to the identification of distinct biological mechanisms and potential therapeutic targets within the subgroups.

We also performed GSEA analysis on the entire set of genes linked to two distinct inflammatory patterns. Our results demonstrated notable variations in certain pathways, particularly in the TGF beta signaling, TNFA signaling via NFKB, P53 pathway, etc (**Figure 10A**). In addition, GSVA results revealed that growth hormone receptor signaling via JAK-STAT, negative regulation of autophagy, acute myeloid leukemia, cell growth, TGF beta signaling, positive regulation of inflammatory response to antigenic stimulus and P53 pathway were activated in the C2 subgroup, which were consistent with the results of KEGG and GSEA (**Figures 10B, C**). Collectively, our findings from both GSEA and GSVA analysis, along with the functional enrichment analysis, provide comprehensive evidence supporting the activation of specific pathways in the C2 subgroup. These findings shed light on the molecular mechanisms underlying the inflammatory patterns observed in this subgroup and may pave the way for the development of targeted therapeutic approaches for AR associated with these activated pathways.

### Therapeutic target prediction

**Figure 11:**
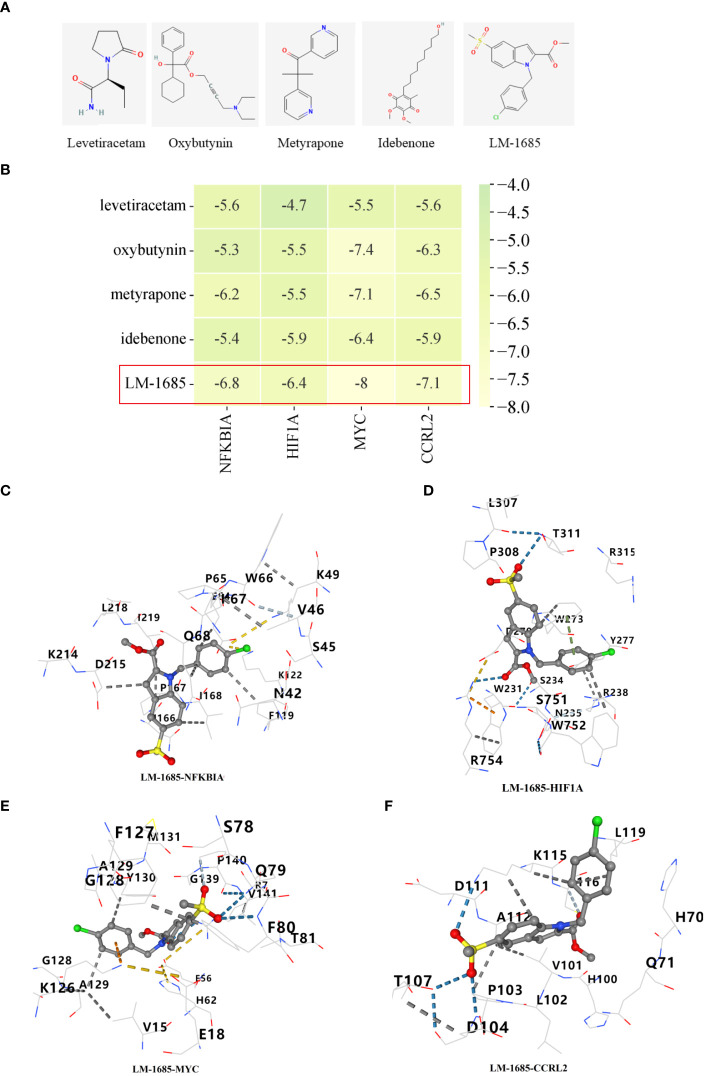
Screening of potential drugs for patients with C1 subtype. **(A)** The chemical structure of the potential small molecule compounds. **(B)** Heat map presenting the lowest binding energy for molecular docking. Molecular docking results of LM-1685 with NFKBIA **(C)**, HIF1A **(D)**, MYC **(E)**, CCRL2 **(F)** targets.

**Figure 12:**
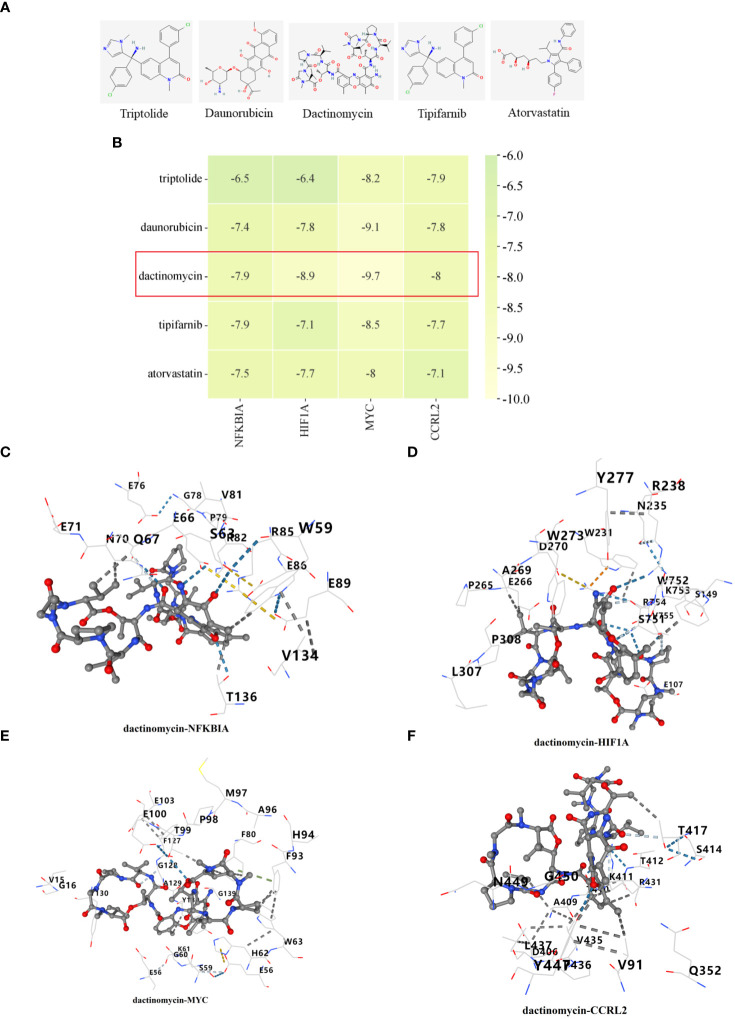
Identification of potential medications for individuals with C2 subtype. **(A)** The chemical structure of the potential small molecule compounds. **(B)** Heat map presenting the lowest binding energy for molecular docking. Molecular docking results of dactinomycin with NFKBIA **(C)**, HIF1A **(D)**, MYC **(E)**, CCRL2 **(F)** targets. These docking studies provided structural insights into the potential inhibitory mechanisms of the candidate compounds against essential proteins involved in AR, and suggest possible therapeutic candidates for further investigation.

Through the utilization of CMap analysis, we aim to uncover potential therapeutic drugs that target AR inflammation-related subtypes. A small molecule compound with a lower CMap score is more likely to possess the capability to treat the AR. As shown in **Supplementary Table S2**, Levetiracetam, oxybutynin, metyrapone, idebenone, and LM-1685 emerged as the five small-molecule drugs with the most favorable CMap score for the C1 subtype (**Figure 11A**). Conversely, triptolide, daunorubicin, dactinomycin, tipifarnib, and atorvastatin ranked as the top five small-molecule drugs with the lowest CMap score for the C2 subtype (**Figure 12A**).

For the molecular docking, we selected five drug candidates and identified the signature genes as NFKBIA, HIF1A, MYC, and CCRL2. The molecular docking results were presented in **Figures 11B**, **12B**. Among these small molecules, LM-1685 and dactinomycin exhibited the strongest binding affinity to the target proteins. Subsequently, we utilized PyMOL software to visualize the molecular docking results, as shown in **Figures 11C–F**, **12C–F**.

## Discussion

AR is a prevalent and persistent respiratory condition that afflicts individuals worldwide. Extensive research conducted by the Global Burden of Disease study has revealed its substantial impact on the well-being of millions, leading to a decline in their quality of life over the past century (31). However, despite significant efforts, the exact causes of AR remain largely unknown and require further investigation. Recently, high-throughput microarray tech and bioinformatics have transformed the study of complex diseases, like allergies. Microarrays have identified many genes in immunotherapy for AR, providing treatment targets (32). The role of the inflammatory response in the development of AR has been suggested (13). To our understanding, this study is the first to investigate and analyze the role of inflammation in the development of AR by identifying and examining IRRGs.

In this research, four primary DIRRGs strongly linked to AR, namely NFKBIA, HIF1A, MYC, and CCRL2, were discovered by machine learning. All these genes are responsible for encoding proteins. NF-κB inhibitor-α (NFKBIA) inhibits the activation of NF-κB and subsequently, signaling in both the NF-κB and EGFR pathways (33). The identification of NFKBIA gene mutations, combined with studies showing an increased occurrence of certain single-nucleotide polymorphisms and haplotypes in different types of cancers, strongly suggests that NFKBIA acts as a tumor suppressor gene (34). At present, there is a lack of reports on the role of NFKBIA in rhinitis. However, the findings from our study are groundbreaking as they demonstrate for the first time a substantial decrease in NFKBIA expression in blood samples from patients with AR. Tumor microenvironments in various types of cancers often experience hypoxia, which leads to the impairment of cytotoxic T cells and facilitates the recruitment of regulatory T cells. Research indicates that hypoxia-inducible factor 1 alpha (HIF1A) is involved in the evasion of the immune system by tumors (35). At the transcriptional level, hypoxia and stabilization of HIF1A are linked to the activation of multiple pathways that regulate inflammation, cell survival, angiogenesis, and metabolism (36). Elevated expression of HIF1A is typically associated with increased patient mortality in various cancer types (37). Previous findings provided evidence that HIF-1α plays a direct role in the onset of allergic airway inflammation (38). In addition, the role of HIF-1α in AR and chronic sinusitis will be crucial, making it a pivotal target for therapeutic interventions in these conditions (39). According to our research findings, the levels of HIF1A in AR blood samples were notably reduced. This indicates a potential crucial role of HIF1A in the development of AR. The MYC gene is a major player in human cancer, making it a key driver in disease development. With its widespread deregulation and significant contribution to cancer initiation, perpetuation, and advancement, targeting MYC is a compelling approach to combat this ailment (40). Currently, there are few reports on MYC’s role in rhinitis. Yet, our study’s findings are groundbreaking, showing a significant increase in MYC expression in AR patients’ blood samples for the first time. Chemokine receptor-like 2 (CCRL2), belonging to the C-C motif chemokine receptor family, is a transmembrane receptor composed of seven domains. It exhibits significant similarity to other chemokine receptors such as CCR1, CCR2, CCR3, and CCR5 (41). The use of Ccrl2-deficient mice in inflammatory disease models highlights the importance of CCRL2 in those conditions (42). In an experiment involving OVA-induced airways hypersensitivity, the removal of CCRL2 gene resulted in impaired transportation of dendritic cells loaded with antigens from the lungs to the mediastinal lymph nodes (43). Currently, few reports discuss CCRL2’s role in rhinitis. Nevertheless, our study’s findings are groundbreaking, revealing a significant decrease in CCRL2 expression in blood samples of AR patients. One of our achievements is the development of a nomogram that combines the four diagnostic markers mentioned above. This nomogram has demonstrated high AUC values and excellent calibration, indicating its accuracy and reliability in diagnosing AR. We anticipate that this tool will be widely used in clinical settings, playing a crucial role in the early detection of AR.

According to reports, there is a strong connection between immune regulation and the occurrence and progression of AR (44, 45). In the present study, the infiltration of immune cells and the results of single gene GSEA indicated that NFKBIA and HIF1A were associated with immune cell infiltration and immune-related pathways in the progression of AR. The development of AR is influenced by an imbalance in the ratio of Tfh2 and regulatory B cells (45). The imbalance of Th1 and Th2 cells has been identified as a critical pathological mechanism in the development of AR (46). The disease pathogenesis of AR may involve the interaction between T and B cells, which is facilitated by the expression of CD23, particularly on switched memory B cells (47). By priming T cells and attracting eosinophils, activated neutrophils can potentially play a role in the development of allergic inflammation observed in AR (48). In our study, we observed a correlation between the expression of NFKBIA and HIF1A and various immune cell types (CD8 T cells, Th1 cells, T helper cells, neutrophils, B cells, and Tcm). The GSEA analysis revealed that the high expression phenotype of NFKBIA and HIF1A was significantly enriched in the B cell receptor signaling pathway. This suggested that NFKBIA and HIF1A may play a role in the regulation of immune processes in AR.

Furthermore, our findings suggested that the identification and characterization of these inflammatory subtypes could have significant implications for the development of personalized therapies for patients with AR. In the present study, the C2 subgroup, which exhibits active TGF beta signaling pathway and P53 pathway, has been shown to play a crucial role in mediating excessive inflammation (49–51). This finding is particularly relevant as both pathways have been implicated in various inflammatory diseases, including rhinitis (52, 53). The activation of these pathways in the C2 subgroup suggested that they may be key players in the development and progression of AR. These findings provided valuable insights into the molecular mechanisms driving inflammation in AR and may have significant implications for the development of personalized therapies. By targeting specific inflammatory subgroups and understanding their unique molecular profiles, we may be able to improve early detection and treatment strategies, ultimately improving the overall management and outcomes of patients with AR.

## Conclusion

Our study underscored the noteworthy influence of the inflammatory response on the progression of AR. We have discovered four DIRRGs that hold promise as biomarkers and therapeutic targets for individuals with AR. Furthermore, based on these DIRRGs, we have identified two molecular subtypes of AR. These findings provide valuable insights into the underlying mechanisms of AR and have the potential to facilitate the development of targeted drug screening and personalized treatment approaches for AR patients.

## Data availability statement

The original contributions presented in the study are included in the article/**Supplementary Material**. Further inquiries can be directed to the corresponding authors.

## Ethics statement

The studies involving humans were approved by the Affiliated Huai’an Hospital of Xuzhou Medical University. The studies were conducted in accordance with the local legislation and institutional requirements. The participants provided their written informed consent to participate in this study.

## Author contributions

JD: Conceptualization, Writing – original draft. KX: Data curation, Formal Analysis, Writing – original draft. DH: Investigation, Methodology, Visualization, Writing – review & editing. SL: Investigation, Methodology, Software, Writing – review & editing. LZ: Formal Analysis, Visualization, Writing – review & editing. SW: Funding acquisition, Project administration, Supervision, Validation, Writing – review & editing. LC: Supervision, Validation, Writing – review & editing.
