# Supplementary material for: Identification of diagnostic signature, molecular subtypes, and potential drugs in allergic rhinitis based on an inflammatory response gene set
Source: Front Immunol. 2024 Feb 26;15:1348391. doi: 10.3389/fimmu.2024.1348391 (PMC10926906; doi:10.3389/fimmu.2024.1348391)
Supplement: Supplementary file 1 [file DataSheet_1.docx]

Table S1 Sequences of primers used qRT-PCR.

| Primers | Forward primer (5' to 3') | Reverse primer (5' to 3') |
| --- | --- | --- |
| NFKBIA | TGGAAGTGATTGGTCAGGTGA | AGGCAAGATGGAGAGGGGTAT |
| HIF1A | ATCCATGTGACCATGAGGAAATG | TCGGCTAGTTAGGGTACACTTC |
| MYC | GCAATGCGTTGCTGGGTTAT | CGCATCCTTGTCCTGTGAGT |
| CCRL2 | CCTGGTTGTGCTTATCCTGGT | AGAATTTTACACATGGGATCGCC |
| β-actin | CTCGCCTTTGCCGATCC | TTCTCCATGTCGTCCCAGTT |

Table S2 CMap analysis identified potential drugs for AR subtypes.

| Subgroups | Score | Name | Description |
| --- | --- | --- | --- |
| C1 | -99.89 | levetiracetam | Calcium channel blocker |
|  | -99.86 | oxybutynin | Acetylcholine receptor antagonist |
|  | -99.79 | metyrapone | Cytochrome P450 inhibitor |
|  | -99.75 | idebenone | Calcium channel modulator |
|  | -99.68 | LM-1685 | Cyclooxygenase inhibitor |
| C2 | -100 | triptolide | RNA polymerase inhibitor |
|  | -99.93 | daunorubicin | RNA synthesis inhibitor |
|  | -99.92 | dactinomycin | RNA polymerase inhibitor |
|  | -99.89 | tipifarnib | Farnesyltransferase inhibitor |
|  | -99.86 | atorvastatin | HMGCR inhibitor |


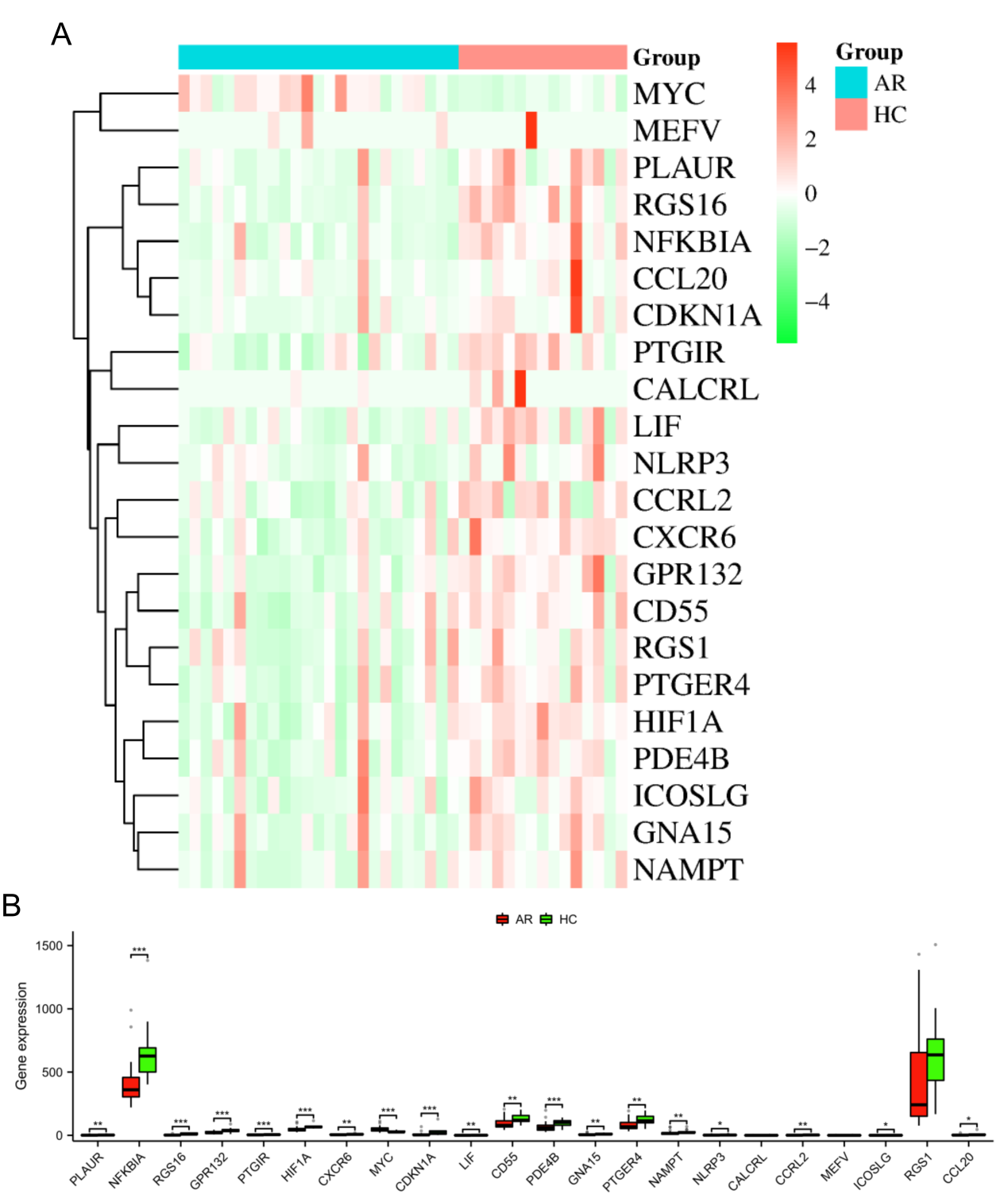


Figure S1 Gene expression levels of IRRGs in AR. Heatmap (A) and box plot (B) depict the expression levels of 22 IRRGs in the AR and HC groups. *p < 0.05, **p < 0.01, and ***p < 0.001.


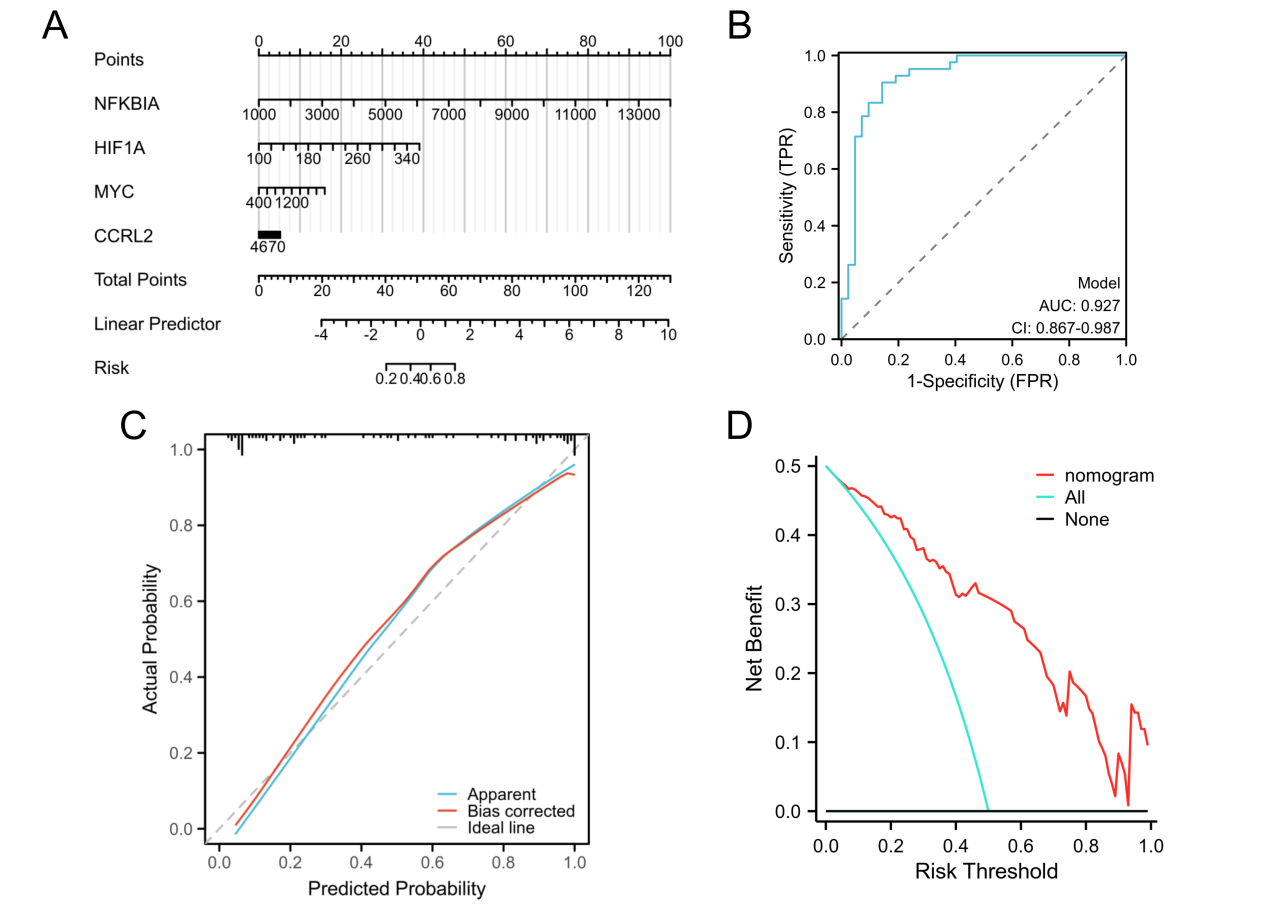


Figure S2 Validation of nomogram by the GSE50223 dataset.
